# Special Issue: Diet and Lifestyle Factors Associated with Allergic Diseases in Early Life

**DOI:** 10.3390/nu17223544

**Published:** 2025-11-13

**Authors:** Remo Frei

**Affiliations:** 1 Division of Respiratory Medicine and Allergology, Department of Pediatrics, Inselspital, University of Bern, 3010 Bern, Switzerland; remo.frei@unibe.ch; 2 Department of Biomedical Research, University of Bern, 3010 Bern, Switzerland; 3 Christine Kühne-Center for Allergy Research and Education (CK-CARE), 7265 Davos, Switzerland

Allergic diseases are among the most prevalent chronic disorders worldwide, and their rising incidence represents an expanding challenge for public health and clinical care. Atopic dermatitis (AD) often marks the first manifestation of an allergy in early childhood and serves as a clinical harbinger of subsequent allergic comorbidities, including food allergies, asthma, and allergic rhinitis. With up to one-third of children now affected by at least one allergic disease, allergy has become a defining feature of modern childhood [1].

Allergic disorders are multifactorial inflammatory conditions driven by T helper type 2 (Th2)-skewed immunity, yet their pathogenesis extends well beyond this paradigm. Genetic susceptibility, epithelial barrier dysfunction, immune dysregulation, and microbial imbalance converge to affect disease onset and trajectory [2].

The dramatic rise in allergic diseases in recent decades cannot be explained by genetics alone. The environmental and lifestyle changes that accompany industrialization are central to this epidemiologic transition [2]. Early-life exposures that promote immune maturation and microbial diversity—such as farm living, animal contact, a larger family size, and diverse diets—have consistently been associated with reduced allergy risk [3]. These protective associations converge into a common biological axis: the developing microbiome [3].

Of particular interest to recent scholarship is greater dietary diversity in infancy, including the early introduction of dairy products, fruits, and vegetables, which has been linked to lower risks of AD and asthma [4].

Diets rich in plant components and dairy enhance the microbial production of short-chain fatty acids, notably butyrate, which promote epithelial barrier integrity and immune tolerance [5,6,7,8,9].

Understanding how diet–microbiome interactions shape allergic trajectories offers a compelling opportunity for preventive interventions targeting the earliest stages of immune development. This Special Issue, ‘Diet and Lifestyle Factors Associated with Allergic Diseases in Early Life’, explores how early environmental, dietary, and lifestyle factors affect the microbiome, immune maturation, and allergy risk in early childhood.

Reliable questionnaire and disease diagnose tools are necessary to conduct studies on environmental lifestyle factors, such as nutrition and its association with allergic diseases. The studies by Pickett-Nairne et al. and Czaja-Bluesa et al. contribute significantly to this topic [10,11].

This study by Pickett-Nairne et al. validated an online pictorial food frequency questionnaire (FFQ), VioScreen-Allergy, designed to assess both general nutrient intake and allergen exposure in women of childbearing age. The FFQ showed good agreement with dietary recall for most nutrients and strong test–retest reliability for nutrients, allergens, and the Maternal Diet Index (MDI). These findings indicate that VioScreen-Allergy might be a feasible and reliable tool that could fill the need for validated dietary questionnaires that can measure overall dietary intake while collecting detailed information on food allergen intake. The use of VioScreen-Allergy might enable us to measure the Maternal Diet Index while conducting randomized controlled trials or during observational studies, and could also be used to assess macro- and micronutrient intake [11].

Cow’s milk protein-induced allergic proctocolitis (MPIAP) remains a diagnostic challenge in pediatric allergy, with few reliable biomarkers reflecting intestinal inflammation. Czaja-Bulsa et al. explored whether fecal eosinophil cationic protein (fECP) and human β-defensin-2 (HBD2) could serve as one of these biomarkers. Their findings show elevated fecal fECP and HBD2 in affected infants, with both markers declining after a milk-free diet, suggesting that they reflect mucosal inflammatory activity. Notably, these biochemical changes lag behind clinical improvement, indicating that mucosal healing may continue after symptoms resolve. However, their diagnostic value remains limited. fECP demonstrated high sensitivity (100%), but poor specificity, risking false-positive results in roughly one out of six unaffected children. Conversely, HBD2 showed low sensitivity, missing about one out of four true cases. Together, these results indicate that fECP and HBD2 are not diagnostic tools, but are rather potential biomarkers for monitoring disease resolution. The study underscores the need for more precise mechanism-based approaches to diagnosing non-IgE-mediated food allergies, integrating molecular and clinical parameters to better guide patient care [10].

Heye et al. examined maternal diet during pregnancy and its influence on AD development in the offspring [12]. Using data from a mother–child cohort, researchers found that higher maternal diet diversity and stronger adherence to a Mediterranean diet were both linked to lower AD risk in offspring. This diet emphasizes fruits, vegetables, legumes, whole grains, fish, and olive oil. The protective effects likely stem from improved maternal immune regulation, reduced inflammation, and beneficial microbiome and epigenetic influences on the child’s immune system. These associations remained significant after adjusting for family history and lifestyle factors. While observational, the findings suggest that promoting dietary variety and Mediterranean-style eating during pregnancy may help prevent childhood atopic dermatitis [12].

Caffarelli et al. highlighted an important interplay between breast feeding and the development of an allergy toward cow’s milk [13]. The review examines why some exclusively breastfed infants might develop allergic reactions to proteins in cow’s milk that are transmitted through breast milk. It describes both IgE-mediated and non-IgE-mediated mechanisms, noting that the latter are often harder to diagnose, since standard allergy tests may fail. The elimination and re-challenge method remains the diagnostic gold standard. The authors discuss the controversial role of maternal milk-free diets, warning that such restrictions may cause nutritional risks without clear evidence of benefit in all cases. They highlight emerging research on the infant gut microbiome, which may influence allergy development and tolerance. Overall, they stress personalized clinical management, careful monitoring, and the need for further studies to clarify the mechanisms and optimize the treatment strategies necessary for allergic infants and their mothers [13].

Finally, Harbottle et al. investigated the timing and motivations behind introducing solid first foods and common allergenic foods [14]. The authors surveyed 42 parents (primarily mothers) of children with documented food allergies in Canada and the United States. They found that approximately 47.6% of parents introduced first foods at age 4–5 months, while 52.4% waited until 6 months or later. Cereals (wheat, oats, rice) were the most common first foods (≈54.8%), followed by vegetables (≈28.6%) and then eggs/fruits. Instruction from healthcare providers was the most influential factor guiding the choice and timing of the introduction of first foods. Despite guideline changes following trials, such as the Learning Early About Peanut (LEAP) trial, that support the early introduction of allergenic foods, the study observed no statistically significant shift in parental practices for children born 2016 or later compared to earlier births [15]. The results suggest that, although providers’ guidance is highly valued by parents, the translation of recent guideline updates into widespread practice remains incomplete [14].

Together, the studies featured in this Special Issue span a broad spectrum, ranging from validated dietary assessment tools to biomarkers of cow’s milk allergy, and from maternal nutrition to infant feeding practices. Collectively, these contributions enhance our understanding of disease monitoring, diagnosis, and potential avenues for intervention. Moreover, they underscore the critical importance of timing, emphasizing that opportunities for prevention may begin as early as pregnancy, extend through the breastfeeding period, and continue into early complementary feeding.

Despite these advances, significant challenges remain. Translating emerging insights into evidence-based prevention strategies demands harmonized methodologies, robust longitudinal cohorts, and integrated multi-omics approaches that are capable of capturing the dynamic interplay between diet, microbes, and the developing immune system.
